# Robust Human Machine Interface Based on Head Movements Applied to Assistive Robotics

**DOI:** 10.1155/2013/589636

**Published:** 2013-12-26

**Authors:** Elisa Perez, Natalia López, Eugenio Orosco, Carlos Soria, Vicente Mut, Teodiano Freire-Bastos

**Affiliations:** ^1^Gabinete de Tecnología Médica, Facultad de Ingeniería, Universidad Nacional de San Juan, 5400 San Juan, Argentina; ^2^Instituto de Automática, Facultad de Ingeniería, Universidad Nacional de San Juan, 5400 San Juan, Argentina; ^3^Departamento de Engenharia Elétrica, Universidade Federal do Espírito Santo, 29075910 Vitoria, ES, Brazil

## Abstract

This paper presents an interface that uses two different sensing techniques and combines both results through a fusion process to obtain the minimum-variance estimator of the orientation of the user's head. Sensing techniques of the interface are based on an inertial sensor and artificial vision. The orientation of the user's head is used to steer the navigation of a robotic wheelchair. Also, a control algorithm for assistive technology system is presented. The system is evaluated by four individuals with severe motors disability and a quantitative index was developed, in order to objectively evaluate the performance. The results obtained are promising since most users could perform the proposed tasks with the robotic wheelchair.

## 1. Introduction

In robotics, navigation is one of the major challenges in control schemes, both in structured as in nonstructured environments. This challenge involves the robot's ability to determine its own position and plan a path towards some specific location, based on the information available of the sensors, the environment structure, and the restrictions imposed by the control scheme. As a special case, the assisted navigation is a hybrid scheme, in which the control is supervised or commanded by a human operator [1]. This area of application is growing every year, due to the prevalence of chronic neurological diseases (as poststroke and others) and population ageing.

Assistive robots are used as locomotion devices [2, 3] such as robotic wheelchairs or mobile robots, objects transportation, and other kinds of assistance. Mobile robots or robotic wheelchairs are automatic transportation devices, capable to navigate through a particular environment with some degree of autonomy and perform specific tasks. Robotic wheelchairs are equipped with adaptable interfaces that allow the use as mobility aid for disabled people. These devices attract the attention of many researchers and help people to reach a major autonomy in their daily life.

People with severe motor disabilities (such as quadriplegia, cerebral palsy, or multiple sclerosis) require specific and complex devices to satisfy their needs. Robotic wheelchairs, assistive robots, and human-computer interface (HCI) are alternative tools that improve their quality of life and independence. HCIs are developed taking advantages of the residual capabilities which are used as input in the control scheme.

There is a wide range of biological signals and voluntary commands that can be used in the HCI, such as electroencephalographic (EEG), electromyographic (EMG), electroculographic (EOG) signals [4–7], inertial sensors [8, 9], and Vision Based Interfaces (VBIs) [10]. VBIs and inertial sensors (IMU) are the most preferred interfaces, because they are noninvasive and can be adapted to head movements, which are the only residual capabilities in severe physical disabilities. For these reasons, there is a lot of effort in the development of better HCIs.

Moreover, the control strategy used in the wheelchair command is a decisive factor to the performance of the overall system, assuring the control of velocity and stability of the wheelchair. Many researchers have developed and reported different control strategies for assistive mobile devices. As an example, the work of Ju et al. [10] describes the design and implementation of a hands-free system for intelligent wheelchair. An interface based on head gestures is developed, based on the fusion of the face detection algorithm with the object tracking algorithm. The control architecture has two control modes, called intelligent control and manual control. However, this work does not show a control law that guarantees that the wheelchair would reach the desired velocities. In [11] a robotic wheelchair for cognitively disabled children is presented. Three HCIs are provided: (i) one based on speech recognition, (ii) a motion interpreter, and (iii) the last one based on visual feedback. The results are very promising since the children were able to guide the wheelchair from the first time. However, none of above works provides a control system which ensures that the wheelchair achieves the desired velocity. Furthermore, this system is not suitable for people with severe brain and spinal injury who do not have recognizable speech and haptic sensing abilities.

In this work, the main objective is to develop an alternative tool of mobility or assistance for people with severe motor disability. We propose a system for these people, designed to command the navigation of a robotic wheelchair. The system combines the information of head orientation provided by a VBI and an inertial sensor. These variables are translated into reference signals for a robotic wheelchair. The proposed system includes the HCI, that allows the user to control the assistive device, and the control algorithms based on the kinematic and dynamic models of the robotic wheelchair in order to regulate the linear and angular velocities. By using specific control algorithms, the navigation results to be safer for the users. In fact, the approach of a complete assistance system is an improvement in the area, because some works that can be found in the literature focus their research only on one of two main areas: the interface with the user [12–15]; or the control system for autonomous navigation [16–18]. The first topics present different HCIs without ensuring a comfortable and safe navigation for the user, because the systems do not include control algorithms in order to avoid abrupt changes of velocity. On the other hand, the papers that emphasize the autonomous navigation do not contemplate the user intention related to the chosen path and his/her intentionality, undermining the sensation of controllability of the wheelchair. For these reasons, this paper proposes a complete system, including a robust (against measurement outliers) HCI and a control scheme designed for wheelchairs navigation, considering their kinematic and dynamic models.

Moreover, the robustness of the HCIs is also improved, because two different interfaces (VBI and IMU) determine the rotation angles of the user's head and the information is combined obtaining a unique estimation with less sensing error. This estimation is carried out by a decentralized Kalman Filter [19]. The luminosity variations in the VBI, uncertainties and outliers of the IMU sensors, constitute the most common problems of the HCIs. In this approach, the use of fusion algorithms provides a unique angle that is translated in velocity references for the robotic wheelchair. The results show an adequate performance and a softer path tracking.

In the special case of disabilities, there is a wide range of tools to measure the requirement of assistive technologies, the level of disability or functional capabilities of the user, and the cognitive state of the patient. However, there is a lack in the criterions to quantitative evaluation of the performance of assistive devices. In this paper, an index was developed, in order to objectively evaluate the performance of the system in experimental sessions with a group of severe physical disabled volunteers. This index constitutes a contribution to the qualitative and quantitative evaluation of the results in assistive technologies.

The paper is organized as follows. In the Section 2 the system is described, including the image processing algorithm, the inertial sensor, and fusion and control algorithms, as well as the proposed evaluation index.  Section 3 presents the experimental results, and finally in Section 4 we discuss the conclusions of this work.

## 2. Materials and Methods

### 2.1. System Overview

**Figure 1:**
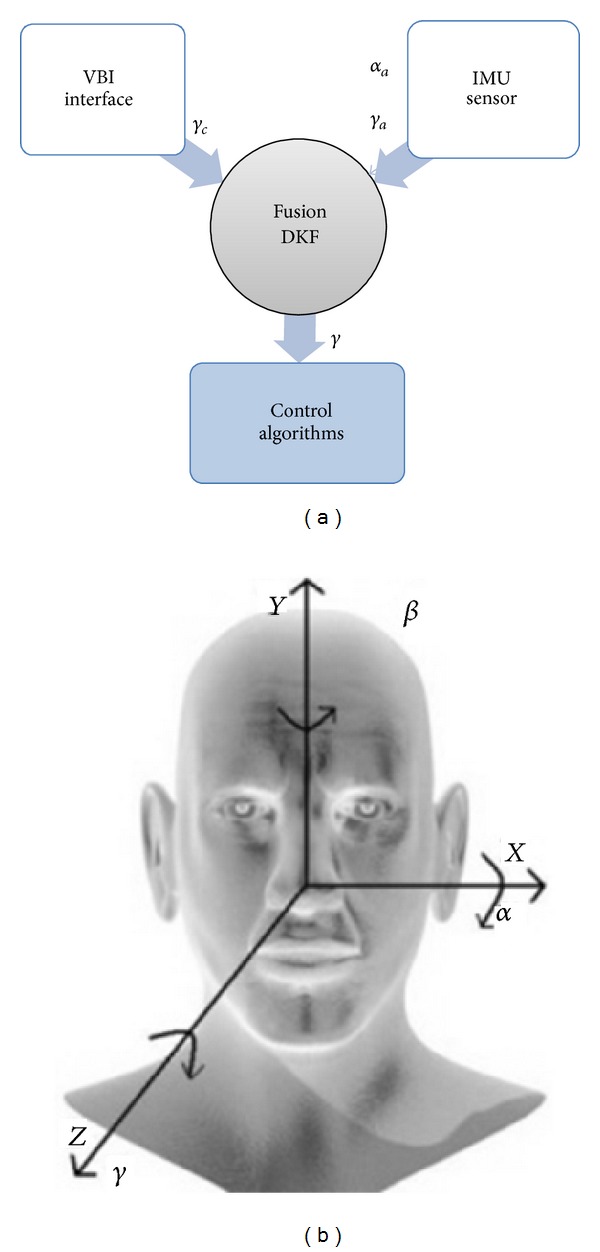
(a) System overview. The DKF fuses the head orientation angles obtained through VBI (*γ*_*c*_) and the IMU sensor (*γ*_*a*_). The estimated angle *γ* is used in the control algorithms. (b) Reference framework associated with the user's head and rotation angles of the head in the 3D space related to the coordinate system.

The proposed system has three main parts: a VBI interface, an IMU interface, and fusion and control algorithms of the wheelchair (Figure 1). More details about the wheelchair used can be found in [5, 18, 20].

The interfaces are noninvasive and of low cost since are used a webcam (resolution: 320 × 240 pixels; frame rate: 10 fps) and an IMU sensor. Both HCIs estimate the rotation angles of the user's head, because they are designed to people with severe disabilities, who cannot mobilize their hands or fingers.

Then, the values of the angles obtained by these two techniques are combined in order to estimate the head's orientation by the Kalman Fusion algorithm. It is important to note that the HCI obtains two parameters from the user head, that is, the orientation angles of head *α* and *γ* in the space, relative to *X* and *Z* axes, respectively (Figure 1). Angle *γ* is estimated by both techniques and the results are combined in the fusion process that improves the estimation. This angle is used to generate angular velocities to command the wheelchair. The angle *α* is estimated only by the accelerometer, and it is used in the calculus of the linear velocity command. Figure 1 shows the overall system and the reference system of the rotation angles of the head.

### 2.2. Vision Based Interface

Artificial vision is used to estimate head movements through face detection [21]. A method for face detection is based on features, which extracts image features and tracks the movement along subsequent images. The commonly used image features are based in regions, skin colour, contours, and landmarks. In this work the skin colour was chosen for detecting and tracking the face, using the colour space YCbCr, because the skin colour is grouped in a precise region of this space [21–23].

The first step of the detection process is the compensation of the illumination. Some authors [22–25] use detection of the skin colour with different techniques. They propose different preprocessing algorithms for obtaining a stable image when there are changes in the illumination. In this work, to address this issue, light compensation technique is employed. With this aim, histogram equalization of the input image is performed. This method improves the quality of the image by increasing the dynamic range of the pixels and enhancing the image contrast.

The second step is the detection of the skin by segmentation in the YCbCr colour space. The original image is first transformed from RGB to YCbCr space by

(1) $$\begin{array}{c} \text{Y}=0.3\text{R}+0.6\text{G}+0.1\text{B},\\ \text{Cr}=0.5+0.4375\text{R}-0.375\text{G}-0.0625\text{B},\\ \text{Cb}=0.5-0.15\text{R}-0.3\text{G}+0.45\text{B}. \end{array}$$

The YCbCr space is selected because its components are less variant to different skin tones [23]. Segmentation is performed by thresholding Cr and Cb components [26]. The threshold values were empirically selected as 130 < Cr < 170 and 70 < Cb < 127.

**Figure 2:**
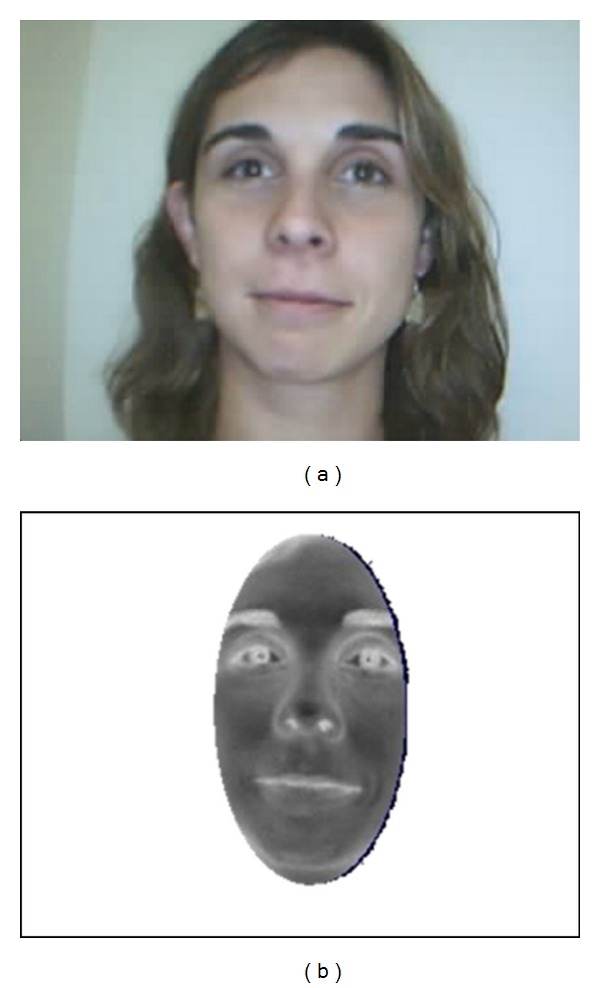
Vision based interface. (a) Image obtained by the webcam interface at a resolution of 320 × 240 pixels. (b) Ellipse ROI image obtained after the skin detection (in YCbCr space) and the ellipse calculation.

The third step is determining the maximum connected skin zone, in order to eliminate the objects on the background. The final step is to calculate an ellipse to localise the face, whose centre matches with the mass centre of the segmented image. This ellipse defines the region of interest (ROI) for extracting the facial features in the luminance component Y of the YCbCr space (Figure 2).

Different techniques to obtain facial features have been presented in the last years; each one of these techniques presents some advantages and some disadvantages. In this work we propose the integration of two methods for facial features extraction and tracking, in order to improve the performance. These two methods are (a) the classic K-means algorithm and (b) the classic normalized correlation (NC). K-means technique is used for determining the centre of a set of features points, while NC is used to identify the eyes' region based on a template of the eyes.

The algorithm proposed can be subdivided into three well defined steps: step (1) is extraction of features points and computation of the centroid of the facial features in the image; step (2) is normalized correlation; step (3) is tracking of the features by combination of the results obtained in the previous steps.

**Figure 3:**
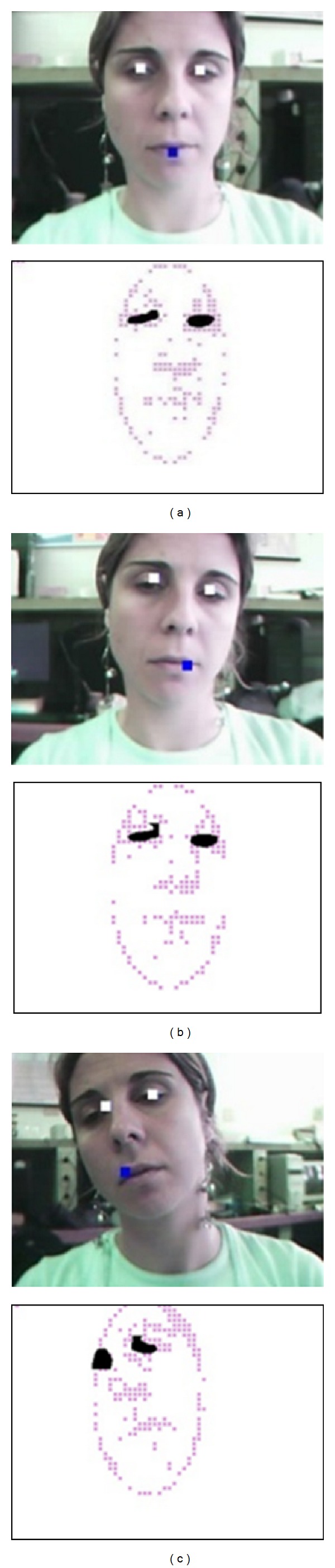
Examples of the facial characteristics detection. Three cases of eyes detection are shown, these case are (a) centred head, (b) head's left-move, and (c) head's right-move.

*(1) Extraction of the Characteristic Points*. Facial features shown in Figure 3 present stronger and brighter contours than the surrounding regions. These characteristics allow the use of the method of the characteristic points [27], which represents a fast and simple method for detection and localization. Feature points selected correspond to contours or corners.

Considering a point *p* and a region surrounding it of 5 × 5 pixels, denominate *Q*, a matrix **C** that represents the gradients in the region can be determined as

(2) $$\begin{array}{c} C=\left[\begin{array}{cc} \underset{Q}{\sum }E_{x}^{2} & \underset{Q}{\sum }E_{x}E_{y}\\ \underset{Q}{\sum }E_{x}E_{y} & \underset{Q}{\sum }E_{y}^{2} \end{array}\right], \end{array}$$

where *E*_*x*_ and *E*_*y*_ are the gradient of each point in the region *Q* in the axes *x* and *y*, respectively. As **C** is symmetric, it can be diagonalized as

(3) $$\begin{array}{c} C=\left[\begin{array}{cc} \lambda_{1} & 0\\ 0 & \lambda_{2} \end{array}\right], \end{array}$$

where  *λ*_1_ and *λ*_2_ are the eigenvalues of the matrix **C**. Through the geometric interpretation of the eigen values of **C**, it can be determined if a pixel represents a corner or not. Since corner is the result of two strong contours, all pixels with intensity value greater than the minor eigenvalue belong to a corner. This way, it is possible to obtain the characteristic points of the image, which correspond to the region of the face that has strong contour (eyes, mouth, eyebrows, etc.).

Facial features need the calculus of the mass centres of the two regions of interest (eyes). These mass centres are obtained by clustering the feature points and discarding points which are not associated with regions of the facial features. With this aim, a simple and efficient K-means algorithm is used.

*(2) Normalized Correlation*. The second step consists of the optimization of feature extraction based on a correlation process. For this purpose, a subimage of 50 × 36 pixels is used. This subimage of a known image of the frontal face is compared with the eyes region on the luminance image using fast correlation to identify the eye location.

*(3) Tracking of the Features*. The average mass centres values of the regions associated with each eyes, obtained in the previous technique, are filtered using a Kalman filter to obtain the location of the eyes. The filter considers a first order kinematics model whose states correspond to the measurement of the centroids. The values of the covariance matrices of the Kalman filter *Q*_Kalman_ and *R*_Kalman_ are 1 × *I*_2×2_ and 5 × *I*_2×2_, respectively. Examples of the image with the correlation and the characteristic points estimated can be observed in Figure 3.

The value of the rotation angle *γ*_*c*_ is obtained using the eyes centroid as

(4) $$\begin{array}{c} \gamma_{c}=\tan^{-1}\frac{y_{1}-y_{2}}{x_{1}-x_{2}}, \end{array}$$

where (*y*_1_, *x*_1_) are the centroid coordinates of the right eye and (*y*_2_, *x*_2_) are the centroid coordinates of the left eye.

### 2.3. IMU Interface

As mentioned in the preceding paragraph an accelerometer is used for estimating two angles of the head orientation in the space. These angles are *γ*_*a*_ (related to the *Z*-axis) and the angle *α*_*a*_ (related to the *X*-axis).

**Figure 4:**
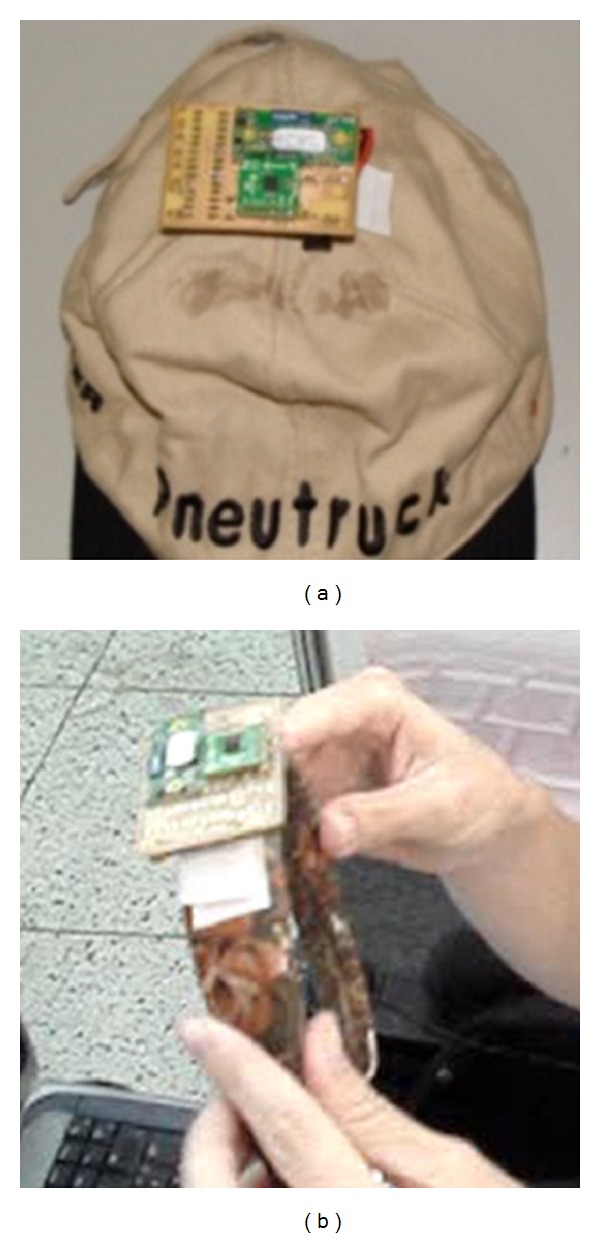
Inertial sensor mounted on a cap or headband. These two accessories were proposed to provide comfort to the user's tastes.

The accelerometer used is the ADXL322j from Analog Devices. This accelerometer requires a voltage supply between 2.4 and 6.0 volts and low power consumption with an average current of 340 *μ*A in operation, and its dimensions are 4 × 4 mm, allowing the use in a discrete and portable assembly. This sensor has two output signals; one of them is for the deviation with respect to *X*-axis, and the other one is for deviation relative to *Z*-axis, which varies linearly with the inclination of the sensor. The accelerometer and a filter stage are implemented on a small board (Figure 4). The microcontroller used is a PIC16F876A manufactured by Microchip. This microcontroller reads the data sensed and transmits them to a Bluetooth communication module OEMSPA311i (ConnecBlue). The data are transmitted via Bluetooth protocol to the computer at 2.4 GHz, where the fusion with the angle estimated by image processing is carried out. The obtained system is small, simple, and of low cost and has lower consumption of power (Figure 4). The device is mounted on a classical cap or a headband.

Once the inertial sensor is placed on the head of the user, the software developed makes a first reading of the inclination angles of the head and these angles are saved for relating all subsequent measurements to these first values. This way, an offset correction is carried out when the assistive device is initialized.

### 2.4. Fusion Algorithm

Estimation tools such as the Kalman filter can be used to combine or fuse information from different sources or sensors for hybrid systems. The Decentralized Kalman Filter (DKF) generates the overall signal estimate by minimizing the variances [19]. The DKF can be considered an algebraic equivalent of the Centralized Kalman Filter (CKF). Theoretically, there is no performance loss in the decentralized system; it delivers the same results as the CKF, but the benefits of the DKF are the modular concept that allows to add more sensors to the system, as needed, and an easier parallel implementation. In this work, fusion is used to decrease the variance of the angle estimations in an optimal way, improving the interface performance [19].

The angular values *γ*_*a*_ and *γ*_*c*_, and their variances obtained by both techniques, are introduced in a DKF, where the angle fusion is carried because the angle *γ*_*a*_ estimated by the accelerometer presents abrupt changes when the user moves the head. These changes could produce undesired movements to the wheelchair when the user is driving it. On the other hand, this sensing technique is more stable than the image processing technique, because the technique based on artificial vision depends on the centroids of the eyes and it is not always detected, due to abrupt changes in the illumination or wide movement of the head. These problems could produce errors in the calculus of the *γ*_*c*_ angle. For this reason, the fusion of the two angles is implemented. Thus it provides an interface with better performance and stability for the navigation of the chair than without using the fusion.

### 2.5. Wheelchair Model

**Figure 5:**
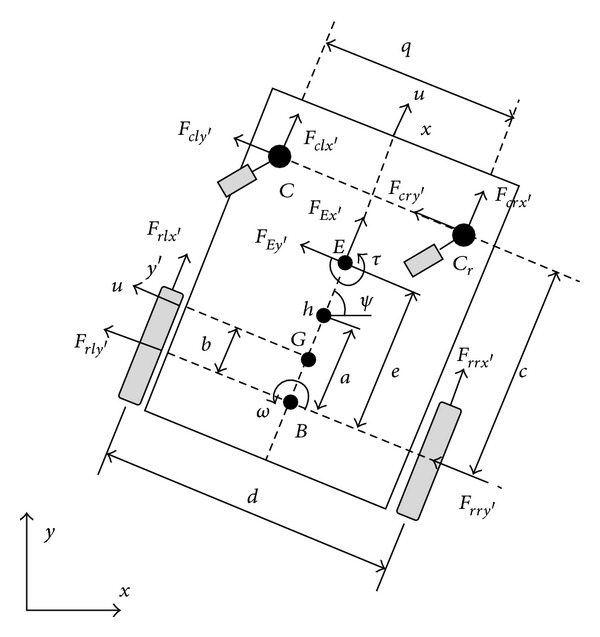
Parameters of the dynamic model of the robotic wheelchair. The relevant parameters are *u* linear velocity, *ω* angular velocity, *G* center of mass, *c* middle point between front wheels, *E* mass center of the user location, *h* : (*x*, *y*) point of interest, *ψ* robot orientation, and *a* distance between *h* and the central point of the virtual axis of the traction wheels.

The control laws used in this work consider the dynamic model developed by de la Cruz et al. [18]. This model is based on the contributions of [28], considering velocity references as inputs. The model of the wheelchair is presented in Figure 5. This figure depicts the wheelchair with the parameters and variables of interest. In the figure, *u* and *ω* are the linear and angular velocities of the wheelchair, respectively, *G* is the center of mass of the wheelchair, *c* is the position of the middle point between the front wheels, *E* is the mass center of the user location, *h* is the point of interest with coordinate *x*, *y* in the *XY* plane, *ψ* is the robot orientation, and *a* is the distance between the point of interest and the central point of the virtual axis linking the traction wheels.

The mathematical representation of the complete model can be seen in the same way of mobile robots and is given by the following.

Kinematic Model:

(5) $$\begin{array}{c} \left[\begin{array}{c} \dot{x}\\ \dot{y}\\ \dot{\psi } \end{array}\right]=\left[\begin{array}{cc} \cos\psi & -a\sin\psi \\ \sin\psi & a\cos\psi \\ 0 & 1 \end{array}\right]\left[\begin{array}{c} u\\ \omega \end{array}\right]+\left[\begin{array}{c} \delta_{x}\\ \delta_{y}\\ 0 \end{array}\right]. \end{array}$$

Dynamic Model:

(6) $$\begin{array}{c} \left[\begin{array}{c} \dot{u}\\ \dot{\omega } \end{array}\right]=\left[\begin{array}{cc} \frac{\theta_{3}}{\theta_{1}}\omega^{2} & -\frac{\theta_{4}}{\theta_{1}}u\\ -\frac{\theta_{5}}{\theta_{2}}u\omega & -\frac{\theta_{6}}{\theta_{2}}\omega \end{array}\right]+\left[\begin{array}{cc} \frac{1}{\theta_{1}} & 0\\ 0 & \frac{1}{\theta_{2}} \end{array}\right]\left[\begin{array}{c} u_{\text{ref}}^{d}\\ \omega_{\text{ref}}^{d} \end{array}\right]+\left[\begin{array}{c} \delta_{u}\\ \delta_{\omega } \end{array}\right]. \end{array}$$

The vector of model parameters and the vector of uncertainties parameters are, respectively,

(7) $$\begin{array}{c} \theta ={\left[\begin{array}{cccccc} \theta_{1} & \theta_{2} & \theta_{3} & \theta_{4} & \theta_{5} & \theta_{6} \end{array}\right]}^{T},\\ \delta ={\left[\begin{array}{ccccc} \delta_{x} & \delta_{y} & 0 & {\overset{-}{\delta }}_{u} & {\overset{-}{\delta }}_{\omega } \end{array}\right]}^{T}. \end{array}$$

The vector ***θ*** was obtained through an identification experiment, which can be found in [18], and the values obtained were

(8) $$\begin{array}{c} \theta_{1}=0.4087,\;\;\theta_{2}=0.1925,\\ \theta_{3}=0.0047,\;\;\theta_{4}=1.0042,\\ \theta_{5}=0.0044,\;\;\theta_{6}=0.8744. \end{array}$$

### 2.6. Control Scheme

The control system here implemented has two well-differentiated stages of control. The first stage is based on the kinematic model of the wheelchair. In this stage, reference velocities are computed as functions of the orientation angles of the head, obtained from the interface.

These reference velocities are the inputs of the second stage, designed according to the dynamic model that generates the control actions to be sent to the robotic wheelchair.

#### 2.6.1. Design of the Kinematic Controller

As was mentioned above, the kinematic controller uses the orientation angles *γ* (estimated by DKF) and *α*. The angle *γ* is used in the angular velocity control law, while both angles *α* and *γ* are used in the control law for linear velocity.

The nonlinear control law for the angular velocity used is

(9) $$\begin{array}{c} \omega_{\text{ref}}=-k_{\omega 1}\tanh\tilde{\psi }, \end{array}$$

where *k*_*ω*1_ is a positive design constant and $\tilde{\psi }=\gamma -\psi$ is the heading error of the robot. The function tanh(·) is used to prevent the saturation of the angular velocity command when high orientation errors exist. The analysis of stability of this law of control is developed in [29].

The control law for the linear velocity was developed in such a way that the velocity is reduced when the robotic wheelchair is manoeuvring; that is, when an orientation error $\tilde{\psi }$ exists. Therefore, the control law for the linear velocity is

(10) $$\begin{array}{c} u_{\text{ref}}=V_{\max}\cos\tilde{\psi }\;\text{if}\;\;\alpha >0,\\ u_{\text{ref}}=0\;\text{if}\;\;\alpha <0. \end{array}$$

This way, the maximum linear velocity is *u*_ref_ = *V*_max⁡_. The maximu velocity *V*_max⁡_ should be defined taking into account both the physics limits of the wheelchair (avoiding the saturation of the actuators), as well as the comfort and safety of the user.

#### 2.6.2. Design of the Dynamic Controller

This controller compensates the wheelchair dynamics, improving the performance of the proposed system. The dynamic controller receives the velocity references from the kinematic controller and generates the linear and angular velocities to be sent to the wheelchair.

The dynamic controller is based on the nominal dynamic of the wheelchair, which represents the estimated medium dynamics, disregarding the uncertainties. This nominal dynamic can be represented as

(11) $$\begin{array}{c} \left[\begin{array}{c} \dot{u}\\ \dot{\omega } \end{array}\right]=\left[\begin{array}{c} \frac{\theta_{3}}{\theta_{1}}\omega^{2}-\frac{\theta_{4}}{\theta_{1}}u\\ -\frac{\theta_{5}}{\theta_{2}}u\omega -\frac{\theta_{6}}{\theta_{2}}\omega \end{array}\right]+\left[\begin{array}{cc} \frac{1}{\theta_{1}} & 0\\ 0 & \frac{1}{\theta_{2}} \end{array}\right]\left[\begin{array}{c} u_{\text{ref}}^{d}\\ \omega_{\text{ref}}^{d} \end{array}\right]. \end{array}$$

From (11) and without considering the uncertainties, the inverse dynamics of the robotic wheelchair can be parameterized as follows:

(12) $$\begin{array}{c} \left[\begin{array}{c} u_{\text{ref}}^{d}\\ \omega_{\text{ref}}^{d} \end{array}\right]=\left[\begin{array}{cccccc} \dot{u} & 0 & -\omega^{2} & u & 0 & 0\\ 0 & \dot{\omega } & 0 & 0 & u\omega & \omega \end{array}\right]\theta , \end{array}$$

which can be rewritten as

(13) $$\begin{array}{c} \left[\begin{array}{c} u_{\text{ref}}^{d}\\ \omega_{\text{ref}}^{d} \end{array}\right]=\left[\begin{array}{cc} \theta_{1} & 0\\ 0 & \theta_{2} \end{array}\right]\left[\begin{array}{c} \dot{u}\\ \dot{\omega } \end{array}\right]+\left[\begin{array}{cccccc} 0 & 0 & -\omega^{2} & u & 0 & 0\\ 0 & 0 & 0 & 0 & u\omega & \omega \end{array}\right]\theta . \end{array}$$

The proposed inverse dynamics control law is

(14a) $$\begin{array}{c} \nu_{\text{ref}}^{d}=G\left(u,\omega ,u_{\text{ref}},\omega_{\text{ref}},{\dot{u}}_{\text{ref}},{\dot{\omega }}_{\text{ref}}\right)\theta , \end{array}$$

where

(14b)G=[σ10−ω2u000σ200uωω],(14c)σ1=u˙ref+ku(uref−u),σ2=ω˙ref+kω(ωref−ω).

### 2.7. Experimental Protocol and Evaluation Index

The performance evaluation of the proposed system follows the Human Activity Assistive Technology (HAAT) model [30]. According to this model, the system to be evaluated comprises not only the assistive device but also the user, the activity carried out by user, and the context where the activity was developed. Therefore, the system is effective if it is useful to the user for achieving the objectives of the proposed tasks.

The objective stated for this assistive technology system is that an individual with motor disabilities can drive the wheelchair to a precise localization. The evaluation of the activity is carried out in two stages. In the first stage the user becomes familiar with the human-computer interface and the navigation of the wheelchair. In this training stage the user performs a free navigation, no longer than three minutes, without any predetermined task. Each user completes four or five free navigations. This first stage is useful not only for the user's training but also for establishing the maximum wheelchair's velocity by taking into account the comfort and safety of each user.

The second stage consists of achieving a final position in the structured environment, while avoiding static obstacles. The described path is subdivided in three steps: *step 1*, the path from the beginning to the obstacles, *step 2*, avoiding the obstacles without colliding, and *step 3*, reaching the final destination. This experiment is carried out five times by each user and the information extracted comprises the time taken to perform the task and how many and which stages the user could reach. Once finalized the task proposed, the user answers an inquiry, in order to obtain the opinion of the user about the assistive technology.

After the experiments with the wheelchair are completed, and with the aim of having a quantitative appreciation of each experiment, we propose the performance index Λ such that 0 < Λ < 1. This index is calculated as

(15) $$\begin{array}{c} \Lambda =\frac{1}{n+1}\overset{n}{\underset{i=1}{\sum }}g_{i}+\frac{1}{n+1}e^{-\;t/h}, \end{array}$$

where *n* is the number of stages in the specific task *t* is the time consumed in seconds, *h* = 150 is a constant that is set in function of the expected time for the task *g*_*i*_ indicates if the stage is complete or not, such as *g*_*i*_ = 1 if the stage's objective is reached, and *g*_*i*_ = 0 otherwise. This index is calculated when at least one stage is completed.

## 3. Results

The results will be presented in two sections: the fusion results and experimental results obtained in several tasks with a group of volunteers. Also, the quantitative index proposed is explained in this section.

### 3.1. Fusion Results

The fusion results were evaluated in normal conditions and also introducing variations in luminosity and abrupt head movements, with the aim to introduce measurements errors and evaluate the performance of the fusion algorithm.

**Figure 6:**
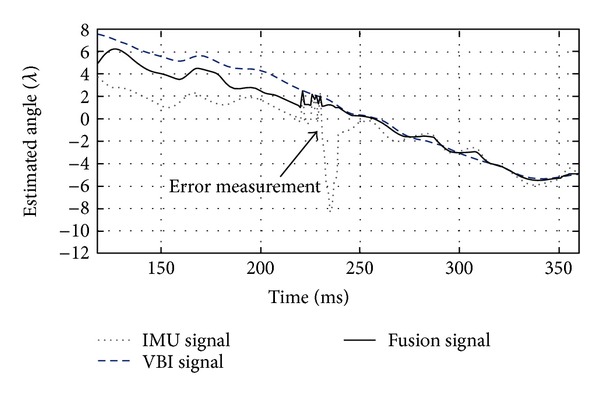
Fusion algorithm and results in laboratory conditions. The angle estimated from the IMU, VBI, and the fusion are tested with nonabrupt and abrupt changes in head's movements. Between 200 and 300 ms, a measurement outlier is filtered by the fusion technique.

Figure 6 shows the time evolution of the of the angle *γ* in estimated signals and the fusion results against a measurement outlier. It is important to note the time evolution between 200 and 300 ms, where abrupt head movements introduce outliers and error measurements that could be translated as an inadequate control signal. The control signal was filtered and smoothed by the fusion algorithm.

### 3.2. Experimental Results

Images of 230 × 240 pixels are captured during the experiments at 10 fps, using a conventional webcam with focal length of 565 pixels. The maximal velocity for the wheelchair, established during the training stage, was 70 mm/s. As we stated previously, the robotic wheelchair used in the experiments has been developed by [4, 20]. The wheelchair is programmed to move forward when the angle *α* is positive (when the head moves ahead), and the wheelchair should stop when the angle *α* is negative (when the head moves back), according to the kinematic control law (10). On the other hand, the wheelchair turns to the right when the angle *γ* is positive (head movements to the right) and it turns left when the angle *γ* is negative (head movements to the left), also according to the kinematic control law (9).

**Figure 7:**
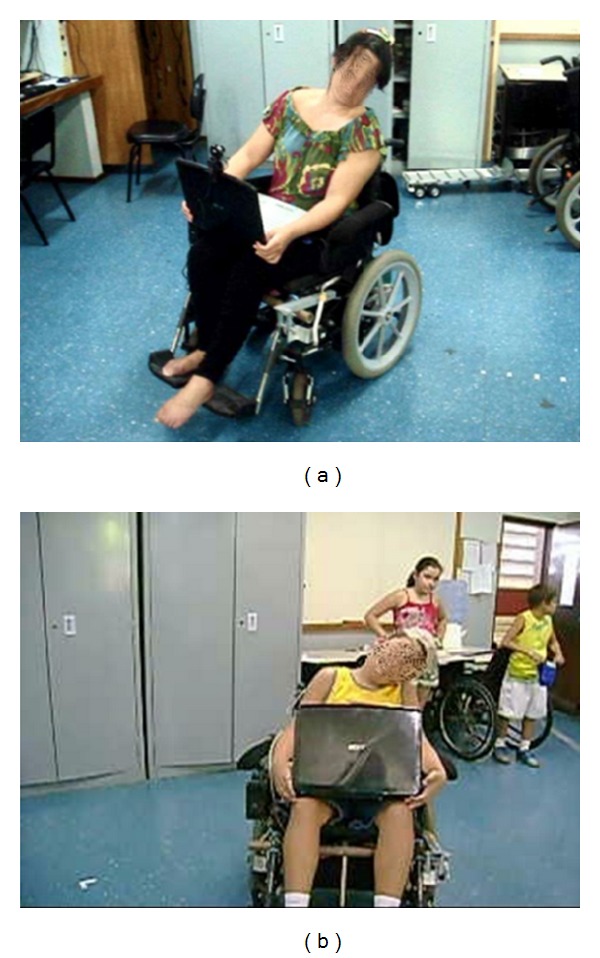
Volunteers with motor disabilities while using the proposed assistive system.

The system was evaluated by four individuals with severe motor disabilities. The users or their parents (in the case of minors) signed the informed consent of the Ethic Committee of the Universidade Federal do Espirito Santo (UFES). Individual A is a fourteen-year-old boy, who has cerebral palsy. Individual B is an eight-year-old girl that has motor anomalies due to a tumour. Individual C is an eleven-year-old boy, with Duchenne muscular dystrophy. Individual D is a quadriplegic thirty five-year-old woman. Some pictures of the volunteers using the robotic wheelchair are shown in Figure 7.

**Figure 8:**
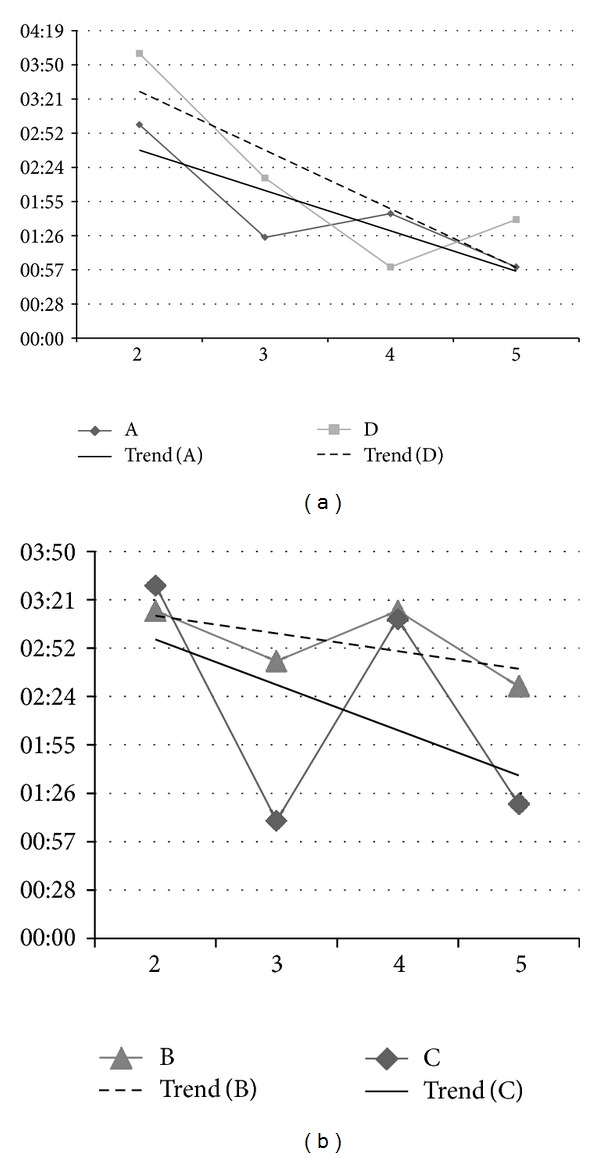
Execution time of experiments. Graphics show total time ([min:sec]) in experiments 2–4 and the downward trend of the times, denoting the training relevance. (a) Adults A and D are presented and (b) childs B and C are exposed.

The data obtained in the experiments are shown in Tables 1, 2, 3, and 4. Columns for each stage are filled with a check symbol (*✓*) if the user completes the stage or with a cross (*✗*) if not. The fifth column shows the total time in the experiment (Figure 8 summarizes these times) and the sixth column shows the index Λ.

In these experimental sessions three stages were accomplished (*n* = 3). Therefore if the user completes, for example, only the first stage, the first term of the index Λ will be equal to 0.25, and the second term will provide a value between 0 and 0.25 according to the consuming time to carry out the task. Therefore, if 0.25 < Λ < 0.5 the user has completed only one stages (being Λ closer to 0,5 when the time spent decreases); if 0.5 < Λ < 0.75 the user has completed two stages (being Λ closer to 0,75 when the time spent decreases); and if 0.75 < Λ < 1 the user has completed three stage (being Λ closer to 1 when the time spent decreases).

The results obtained from the questionnaire can be summarized as follows.

 Individual A: he expressed that commanding the wheelchair is easy and comfortable.
 Individual B: the navigation with robotic wheelchair with the interface, although was easier, she felt unsafe during the first experiments.
 Individual C: he found difficulty in the experiments with the robotic wheelchair. It was hard for him to learn the movements of its head to command the wheelchair.
 Individual D: regarding the robotic wheelchair, she expressed that the systems are comfortable, reliable, and easy to use.

It is important to note that individuals B and C are kids and they can be intimidated by the new interface.

**Figure 9:**
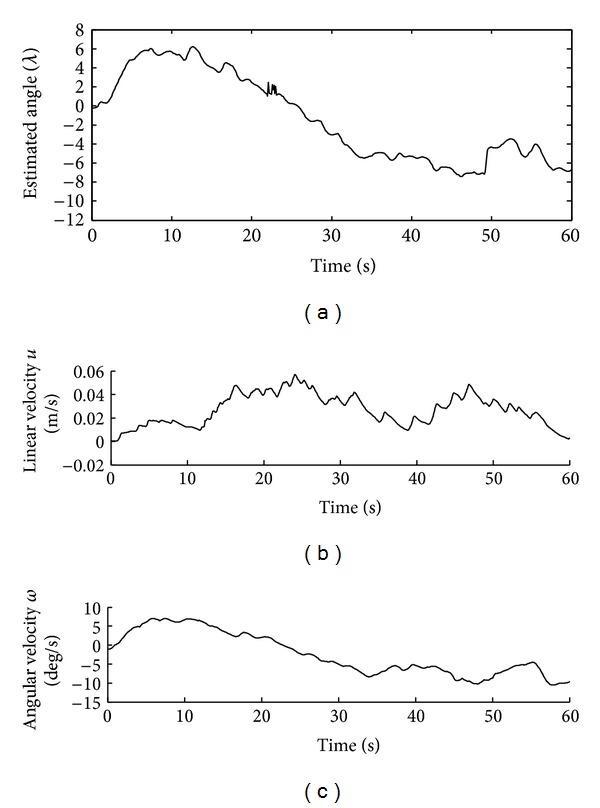
Time evolution of the linear and angular velocities of the wheelchair. The perturbations in the estimated angle are compensated by the control law.

**Figure 10:**
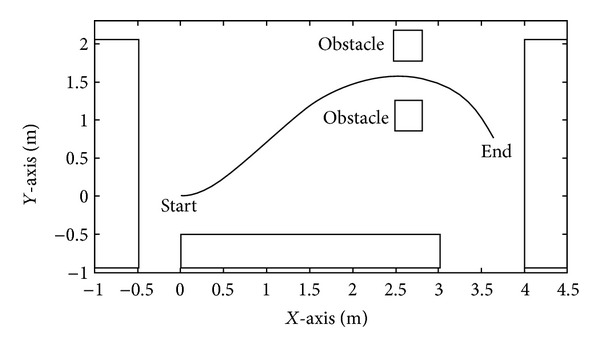
Path described by the wheelchair commanded by the linear and angular velocities of Figure 9.

In Figures 9 and 10, the results of the fourth experiment of one individual (D in this case) are shown. The evolution of the angle *γ* is observed at the top of Figure 9, suited by the angular and linear velocity commands for the wheelchair. Finally the path followed by the wheelchair is shown in Figure 10.

## 4. Conclusions 

In this work an assistive technology system for people with severe motor disabilities was presented, as an alternative tool for locomotion and people assistance. This system has been evaluated by people with severe disabilities, with acceptable performance quantified by the proposed index.

The assistive system uses a combination of VBI and IMU sensors to estimate the pose estimation of the user's head. The pose parameters are combined by a Kalman Filter Fusion algorithm in order to avoid uncertainties, outliers, and error measurements. The fusion process implemented for the *γ* angle improves its performance, obtaining a better estimation of this angle. Additionally, it provides redundancy to the system, which increases the safety for the users. These parameters are used as reference inputs for controlling the navigation of a robotic wheelchair.

Several experiments have been performed in indoor environments with people with severe disabilities. Most users expressed through the inquiry that the control of the robotic wheelchair through the movements of the head is easy and intuitive. Moreover, they pointed out that the navigation is smooth and comfortable. This characteristic of the proposed assistive system is due to the kinematic controller along with the dynamic compensation implemented on-board the wheelchair.

From the results obtained in the experiments, it can be seen that all users, in at least three attempts, reached the objective, leading the chair to the established final position, while avoiding static obstacles. These results are promising because all users could command the wheelchair by using the interface to generate velocity commands, with little training. The time spent to carry out the task decreases while the user acquires more skills and familiarity with the system, which is an important characteristic in the evaluation of this assistive technology. Since the experiments were carried out in an environment similar to a work office and the illumination was not controlled, it is possible to infer that the developed technology can be used to provide autonomy in the locomotion of disabled people. Regarding the performance of the interface itself, it was observed that all users were able to guide the wheelchair in a smooth and safe way for them, without abrupt changes in speed and rotations. This desirable performance was obtained for two reasons, the fusion process implemented for the angle *γ* and the implementation of the control algorithm with dynamic compensation.

Finally, a quantitative index of performance was proposed, with the aim of standardizing the evaluation of the assistive technologies.

## Figures and Tables

**Table 1:** Results of the experiment. User A.

User A	Stage 1	Stage 2	Stage 3	Time (min:sec)	Λ
Exp. 1	*✓*	*✗*	*✓*	2:30	0,592
Exp. 2	*✓*	*✗*	*✓*	3:00	0,573
Exp. 3	*✓*	*✓*	*✓*	1:25	0,892
Exp. 4	*✓*	*✓*	*✓*	1:45	0,874
Exp. 5	*✓*	*✓*	*✓*	1:00	0,918

**Table 2:** Results of the experiment. User B.

User B	Stage 1	Stage 2	Stage 3	Time (min:sec)	Λ
Exp. 1	*✗*	*✗*	*✗*	—	—
Exp. 2	*✓*	*✗*	*✗*	3:15	0,318
Exp. 3	*✓*	*✓*	*✓*	2:45	0,833
Exp. 4	*✓*	*✓*	*✗*	3:15	0,568
Exp. 5	*✓*	*✓*	*✓*	2:30	0,842

**Table 3:** Results of the experiment. User C.

User C	Stage 1	Stage 2	Stage 3	Time (min:sec)	Λ
Exp. 1	*✓*	*✓*	*✗*	3:45	0,556
Exp. 2	*✓*	*✗*	*✗*	3:30	0,312
Exp. 3	*✓*	*✓*	*✓*	1:10	0,907
Exp. 4	*✓*	*✓*	*✓*	3:10	0,820
Exp. 5	*✓*	*✓*	*✓*	1:20	0,897

**Table 4:** Results of the experiment. User D.

User D	Stage 1	Stage 2	Stage 3	Time (min:sec)	Λ
Exp. 1	*✗*	*✗*	*✗*	—	—
Exp. 2	*✓*	*✗*	*✓*	4:00	0,550
Exp. 3	*✓*	*✓*	*✓*	2:15	0,852
Exp. 4	*✓*	*✓*	*✓*	1:00	0,918
Exp. 5	*✓*	*✓*	*✓*	1:40	0,878
